# Evaluation of continuous aspiration of subglottic secretions in prevention of microaspiration during general anesthesia: a randomized controlled pilot study

**DOI:** 10.3325/cmj.2022.63.553

**Published:** 2022-12

**Authors:** Boris Mraovic, Brian Hipszer, Channy Loeum, Angelo Andonakakis, Jeffery Joseph

**Affiliations:** 1 Department of Anesthesiology & Perioperative Medicine, University of Missouri, Columbia, MO, USA; 2 Edwards Lifesciences, Irvine, CA, USA; 3 Thomas Jefferson University, Philadelphia, PA, USA; 4 Cooper University, Camden, NJ, USA

## Abstract

**Aim:**

To assess the difference between endotracheal tubes (ETT) with continuous suction of subglottic secretions (CASS) and standard ETT in preventing secretions movement from the pharynx into the trachea, past the inflated cuff during general anesthesia.

**Methods:**

This randomized, controlled trial enrolled 50 patients who underwent general anesthesia for elective abdominal surgery lasting longer than two hours. They received either ETT with CASS: Teleflex ISIS HVT (GISIS, n = 17) or Mallinckrodt TaperGuard Evac (GEvac, n = 17), or ETT without suction: Mallinckrodt Intermediate Hi-Lo (GStand, n = 16). Methylene blue dye solution (10 mL) was delivered into the hypopharynx every 60 minutes. Subglottic secretions were continuously suctioned. Fiberoptic bronchoscopy was performed every 20 minutes and during tracheal extubation to evaluate the dye location.

**Results:**

The groups did not differ in age, sex, body mass index, race, American Society of Anesthesiologists status, and surgery type. Dye migrated past the inflated cuff into the distal trachea in no patient with ETT with CASS and in 13% of patients with standard ETT. On tracheal extubation, dye migrated into the distal trachea more often in the GStand group (56%), compared with the GEvac (13%) and GISIS group (29%) (*P* = 0.045). The GISIS group had 26 ± 19 mL of secretions suctioned from above the inflated cuff, while the GEvac group had 13 ± 10 mL (*P* = 0.05).

**Conclusion:**

Compared with standard ETT, ETT with CASS efficiently removed secretions during general anesthesia, prevented secretions from migrating past the inflated cuff, and significantly reduced the amount of secretions that reached the distal airways on tracheal extubation.

**ClinicalTrial.gov identification number:**

NCT01386879

Postoperative pulmonary complications (PPC) commonly lead to morbidity and mortality in patients managed with general anesthesia for major surgery. In a systematic review of PPC after non-cardiothoracic surgery, the average incidence was 3.4% with a range from 1% to over 40% (1). Elderly and diabetic patients had the highest incidence. The incidence in abdominal surgery was 14.2% (1). A prospective multicenter study with a heterogeneous surgical population of almost 2500 patients revealed a 5.0% incidence of PPC; with a high mortality in patients who developed PPC (2). Patients with evidence of microaspiration have about three times higher incidence of postoperative pneumonia compared with patients without microaspiration (40% vs 12%), with mortality rate of 19.2% if they develop pneumonia (3).

Endotracheal tubes (ETT) with continuous aspiration of subglottic secretions (CASS) are recommended in intensive care unit (ICU) patients requiring prolonged mechanical ventilation to prevent ventilator-associated pneumonia (4). A meta-analysis showed almost a 50% reduction of ventilator-associated pneumonia in patients receiving ETT with CASS in the ICU compared with standard ETT (5). It is unknown if using an ETT with CASS intraoperatively reduces the incidence of PPC in patients undergoing prolonged general anesthesia, and are extubated at the end of surgery.

We hypothesized that a subglottic suction ETT with CASS would effectively remove secretions that accumulate above the inflated cuff and decrease the volume of secretions aspirated into the distal trachea during general anesthesia with mechanical ventilation and during tracheal extubation. The primary aim of the study was to evaluate whether there is a difference between three types of ETT in preventing the movement of methylene blue dye from the pharynx into the trachea, past the inflated cuff. The secondary objectives were to evaluate whether there is a difference in the volume, pH, and bacterial load of the secretions aspirated from above the subglottic suction ETT cuff.

## PATIENTS AND METHODS

### Study design

This prospective, single-blinded, randomized controlled trial was approved by the Thomas Jefferson University Institutional Review Board. The study was conducted in accordance with the principles of the Declaration of Helsinki. All procedures were performed at Thomas Jefferson University, a single-center tertiary academic medical hospital, between July 2011 and October 2012. The study was terminated after 50 patients because enrollment was slower than anticipated. Written informed consent was obtained from all patients before any study procedures were performed.

### Participants

The study enrolled 50 adults with American Society of Anesthesiologists (ASA) physical status (PS) I – III scheduled to undergo general anesthesia with an ETT for elective abdominal surgery with an anticipated duration longer than two hours. Exclusion criteria were <18 years of age, anticipated difficult airway, pregnancy, history of allergic reaction to methylene blue, methemoglobinemia, glucose-6-phosphate-dehydrogenase deficiency, chronic obstructive pulmonary disease, recent pneumonia (<6 months before surgery), and hypoxemia (hemoglobin oxygen saturation <90% room air or on O_2_).

### Randomization

Participants were randomized into one of three groups. ETT internal diameter ID and surgery type were considered when assigning the type of ETT to use. As recruitment was being performed while the study was ongoing, covariate adaptive randomization was used to ensure a balance of the ETT internal diameter (ID) distribution and surgery type across the three groups. Two groups received an ETT with a subglottic suction port above the cuff: the group GISIS (n = 17) received Teleflex ISIS HVT ETT (Teleflex, Research Triangle Park, NC, USA) and the group GEvac (n = 17) received Mallinckrodt TaperGuard Evac (Covidien, Mansfield, MA, USA). The standard group GStand (n = 16) received a Mallinckrodt Intermediate Hi-Lo ETT with a high volume-low pressure cuff without a suction port (Covidien). The ETT sizes were based upon the participant’s sex and height to ensure the same distribution among all three groups. For men with height <173 cm, ETT 7.5 mm ID was used, for height 173 to 195 cm – ETT 8.0 mm ID, and for height >195 cm – 8.5 mm ID. For women with height <152 cm, ETT size 6.5 mm ID was used, for height 152 to 173 cm – 7.0 mm ID, and for height higher than 173 cm – ETT 7.5 mm ID. Types of surgery were categorized as open or laparoscopic to control for a potential higher aspiration risk in laparoscopic surgeries because of increased abdominal pressure. Patients were recruited and assigned a group on a rolling basis since the investigators did not have control over when the patient's procedure was scheduled. The type of surgery was determined by the patient’s care team and not assigned by the research personnel. ETT type was assigned so that surgery types and ETT IDs were balanced across the three groups.

### Management of anesthesia and recordings

All participants received a standardized induction of general anesthesia. No premedication or antisialagogues were given. After preoxygenation by a face mask and 100% oxygen for 3 minutes, fentanyl 100 µg, propofol 2 mg/kg, and rocuronium 0.6 mg/kg IV were given. All patients were intubated in the supine/sniffing position by experienced faculty anesthesiologists who were investigators in the study. A MAC 3 blade was used, but a stylet for ETT was not used for the first attempt at intubation in all cases. Changing blade type for a second attempt was allowed if needed. After intubation, ETT cuff pilot balloon was connected to a pressure transducer (TrueWave, Edwards Lifesciences, Irvine, CA, USA) for continuous pressure monitoring. Cuff pressure was maintained within the target range (20 ± 2 mm Hg) throughout the study in all three groups, as it is recommended as the safe cuff pressure. Breath sounds were auscultated at the sternal notch to confirm an airtight ETT cuff seal. The tip of ETT was adjusted by fiberoptic bronchoscopy (FOB) to be 2 cm above the carina. General anesthesia was maintained with sevoflurane 2% in air/oxygen 50%, additional doses of rocuronium to keep TOF 1-2 twitches, and fentanyl per the anesthesia team.

In patients who received ETT with CASS, the suction port was connected to a Luken’s trap, and a continuous low suction pressure at -20 cm H_2_O was applied (CASS Suction Regulator Model 3720, Boehringer Labs, Phoenixville, PA, USA) until tracheal extubation. A higher suction pressure (-100 cm H_2_O) was applied once every 20 minutes for 10 seconds starting 20 minutes after intubation. CASS port patency was checked every 20 minutes by visualization of secretion movement with FOB. The suction channel and port were cleared using 10-mL syringe and positive pressure, as needed. Ten milliliters of methylene blue solution (2.5 mL of 1% methylene blue and 7.5 mL of saline), used to determine whether the secretions would go beyond the cuff, were delivered into the hypopharynx using a flexible suction catheter every 60 minutes; starting 20 minutes after intubation.

The presence of the blue dye was video-recorded every 20 minutes using FOB. General anesthesia was maintained with volatile anesthetic and with additional doses of rocuronium and fentanyl. Peak airway pressure and patient’s position on the operating room table were recorded every 20 minutes. At the end of the surgical procedure in the supine position, neostigmine 50 µg/kg with glycopyrrolate 10 µg/kg IV was given for neuromuscular blockade reversal, and secretions were suctioned from the hypopharynx, oropharynx, and mouth before ETT removal. The ETT lumen and distal trachea lumen were not suctioned (standard of care at our hospital). FOB was placed inside of the ETT lumen immediately before tracheal extubation. The cuff was deflated and the tracheal lumen was observed for evidence of aspiration of secretions and blue dye. Patients assigned to GStand had the same procedures performed except that there was no suction connected to the ETT, and secretions were not collected in a Luken’s Trap. Patients were observed in the post-anesthesia care unit (PACU) for one hour to record vital signs, supplemental oxygen use, and respiratory complications. The same variables were recorded 24 hours after leaving the PACU.

### Outcome measures

Two investigators experienced in FOB (BM, JIJ) evaluated the video recordings of all FOB in a blind fashion without knowing which ETT was used. The presence of blue dye and secretions was sought in three locations: the trachea below the vocal cords and above the inflated ET cuff; the distal trachea below the cuff and above the carina; and the bronchi distal to the carina. The presence of dye was graded from 0 to +3 (0 – no dye, 1– up to 15 mm, 2 – 15 to 30 mm, and 3 – larger than 30 mm).

### Statistical analysis

This is a pilot study. The sample size was not calculated. We recruited 10 roll-in patients to confirm feasibility of the study and accuracy of the data. Normality of distribution was assessed with the Anderson-Darling test. Parametric variables are presented as mean ± standard deviation. Differences between the three groups were evaluated with ANOVA with *P* < 0.05 considered significant. Non-parametric (ordinal and nominal) variables are presented as frequencies per variable category. Differences in the frequency distribution were tested by constructing N × M contingency tables, where M is number of groups and N is number of categories for the given variable and tested using the χ^2^ test. Here, *P* < 0.05 would reject the null hypothesis that the table is independent in each dimension at the 95% confidence level. The analyses were performed with Matlab R2013a (Mathworks, Natick, MA, USA).

## RESULTS

**Figure 1:**
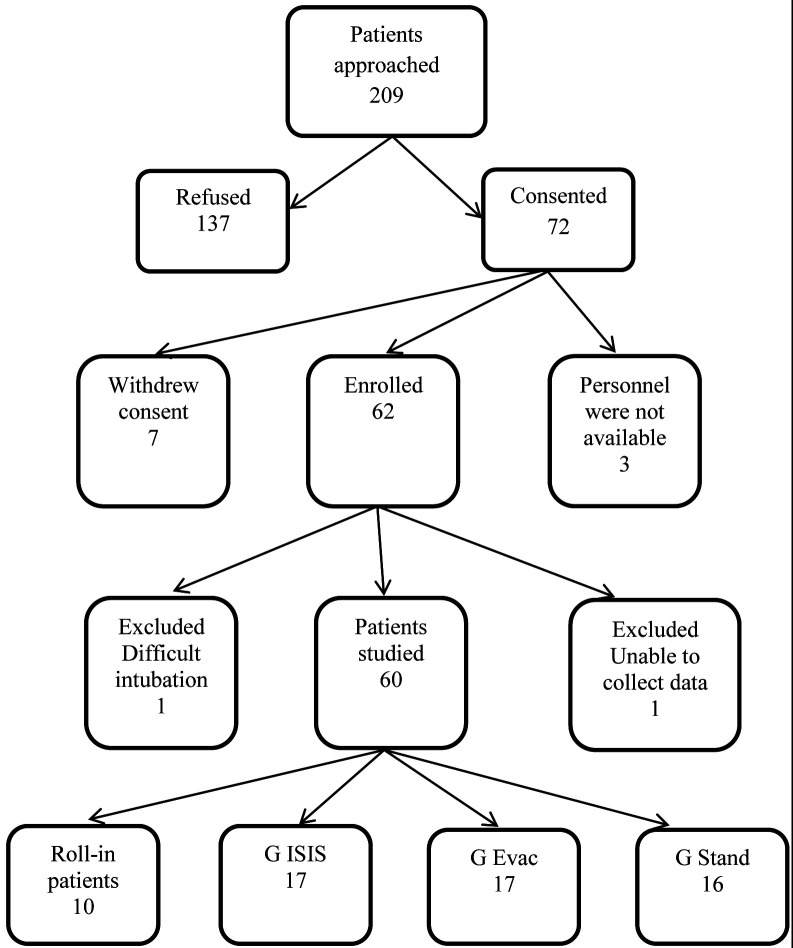
Flow diagram of patients’ distribution.

All patients completed the study. Two of the enrolled patients were excluded (difficult tracheal intubation and equipment failure) (Figure 1).

The groups did not significantly differ regarding demographics, surgery, intubation, and ETT data (Table 1 and Table 2). Although the standard ETT group received less methylene blue, the amount was not significantly different among groups (GStand 24 mL vs GEvac 33 mL and GISIS 33 mL, *P* = 0.167; Table 1). The scheduled administration of the dye was withheld in four GStand patients due to an outflow of dye from the mouth and nose, likely because the standard ETT did not have a suction port and the dye accumulated within the pharynx. There was no point of giving additional dye when it was already leaking out. The majority of instilled dye remained in the pharynx above the soft tissues of the glottis and vocal cords in all patients. Dye passed through the glottis to accumulate above the inflated ETT cuff in 94% of the patients in the GStand, 94% of the patients in the GEvac, and 77% of the patients in the GISIS group (*P* = 0.037) (Table 3).

**Table 1:** Demographics and surgery data

Characteristics	G ISIS* N = 17	G Evac* N = 17	G Stand* N = 16
Sex (F/M)	7/10	7/10	8/8
Age (year)	55.5 ± 15.0	53.9 ± 8.6	54.6 ± 12.9
Height (cm)	170.4 ± 9.9	173.2 ± 9.7	167.4 ± 14.2
Weight (kg)	76.8 ± 13.8	85.9 ± 15.6	76.1 ± 17.32
BMI (kg/m^2^)	26.2 ± 4.1	28.4 ± 3.6	26.8 ± 4.4
ASA status			
I	2	0	2
II	9	12	7
III	6	5	6
Smoker	1	1	3
Surgery type			
open	9	9	8
laparoscopic	8	8	8
Body position			
supine	6	7	7
Trendelenburg	9	6	3
reverse Trendelenburg	1	3	5
lithotomy	1	1	1
Anesthesia duration (h)	3.94 ± 1.54	3.78 ± 1.68	3.17 ± 1.32
Intubation duration (h)	4.07 ± 1.54	3.92 ± 1.68	3.35 ± 1.33
Volatile anesthetic			
desflurane	4	1	1
sevoflurane	13	16	15
Methylene blue given (mL)	32.9 ± 14.5	32.6 ± 17.2	24.4 ± 9.6

*GISIS – Teleflex ISIS HVT tube; GEvac – Mallinckrodt TaperGuard Evac tube; GStand –Mallinckrodt Intermediate Hi-Lo tube ETT without suction.

**Table 2:** Endotracheal tubes and intubation data*

Characteristics	G ISIS N = 17	G Evac N = 17	G Stand N = 16	P value
ETT size (mm)	7.6 ± 0.5	7.5 ± 0.5	7.4 ± 0.6	0.590
Time to intubate the trachea (min)	0.85 ± 0.31	0.83 ± 0.27	0.73 ± 0.29	0.441
ETT depth (cm)	22.6 ± 1.5	22.3 ± 1.7	21.5 ± 1.9	0.286
Number of intubation attempts				
1	14	15	15	0.664
2	2	2	1	
3	1	0	0	
Insertion into the trachea				0.235
easy	11	15	14	
moderate	4	2	2	
difficult	2	0	0	
use of stylet	2	2	1	0.832
Intubation Cormack-Lehane grade view				0.334
I	12	14	11	
II	4	3	2	
III	1	0	3	
Distance between ETT distal end and the carina (mm)	30.8 ± 6.5	26.8 ± 5.5	26.0 ± 6.8	0.067
Cuff pressure (mmHg)	21.2 ± 2.7	20.2 ± 1.8	21.2 ± 2.5	0.389

*Abbreviations: GISIS – Teleflex ISIS HVT tube; GEvac – Mallinckrodt TaperGuard Evac tube; GStand –Mallinckrodt Intermediate Hi-Lo tube ETT without suction; ETT – endotracheal tube.

**Table 3:** Intraoperative bronchoscopy data*^†^

Grade	G ISIS N = 17	G Evac N = 16*	G Stand N = 16	P value
Dye below vocal cords above the inflated ETT cuff				0.037*
0	4	1	1	
1	2	8	1	
2	10	6	12	
3	1	1	2	
Dye in distal trachea below the inflated ETT cuff above carina				0.367
0	17	16	14	
1	0	0	1	
2	0	0	1	
3	0	0	0	
Dye below the carina in the bronchi				0.349
0	17	16	15	
1	0	0	0	
2	0	0	1	
3	0	0	0	
Ischemia of tracheal mucosa below the vocal cords above the inflated ETT cuff				0.941
0	0	0	0	
1	6	5	7	
2	9	9	8	
3	2	2	1	
Blood below the vocal cords above the inflated ETT cuff				0.824
0	5	6	7	
1	9	7	7	
2	2	3	2	
3	1	0	0	
Secretions below the vocal cords above the inflated ETT cuff, external to the ETT				0.368
0	0	2	3	
1	13	12	9	
2	4	2	4	
3	0	0	0	
Secretions in the distal trachea below the inflated ETT cuff above the carina, external to the ETT				0.377
0	5	3	3	
1	6	11	6	
2	5	2	5	
3	1	0	2	
Secretions below the carina in the bronchi				0.673
0	9	11	11	
1	7	4	3	
2	0	1	1	
3	1	0	1	
Secretions within the ETT lumen				0.388
0	0	1	0	
1	8	7	6	
2	7	6	4	
3	2	2	6	

*Abbreviations: GISIS – Teleflex ISIS HVT tube; GEvac – Mallinckrodt TaperGuard Evac tube; GStand –Mallinckrodt Intermediate Hi-Lo tube ETT without suction; ETT – endotracheal tube.

†One bronchoscopy recording in the GEvac group was corrupted and not able to be evaluated.

In the suction ETT groups, no secretions or dye migrated past the inflated cuff into the distal trachea. In contrast, the cuff of the standard ETT (without CASS) developed folds or invaginations that allowed a small amount of dye and secretions to channel downward along the trachea wall. Despite proper cuff inflation, dye migrated into the distal trachea of 13% (2/16) of the standard ETT patients, but the difference among the groups was not significant (*P* = 0.367). The dye that passed the inflated cuff migrated into the bronchi of one GStand patient (Table 3). The GISIS group had, on average, twice the total volume of secretions suctioned per patient compared with the GEvac group (26 ± 19 mL from 10 patients vs 13 ± 10 mL from 12 patients), but the result was borderline non-significant (*P* = 0.055) (Table 4). The groups did not differ in the pH of secretions and the bacterial load or type of bacteria (Table 4).

**Table 4:** Intraoperative secretions*

Characteristics	G ISIS N = 17	G Evac N = 17	P value
Total secretion volume (mL)	26 ± 19	13 ± 10	0.055
Total secretion weight (g)	20 ± 15	10 ± 9	0.075
Average secretion volume per sample collected every 20 min (mL)	9 ± 4	7 ± 3	0.232
Average secretion weight per sample (g)	7 ± 4	5 ± 4	0.410
Average secretion pH	7.3 ± 0.4	7.4 ± 0.6	0.712
Gram stain any organism	13	13	NS
positive	5	2	0.448
negative	0	0	
indeterminate	0	0	
positive and negative	7	8	
positive and indeterminate	0	1	
negative and indeterminate	0	0	
all types	1	2	

*Abbreviations: GISIS – Teleflex ISIS HVT tube; GEvac – Mallinckrodt TaperGuard Evac tube; GStand –Mallinckrodt Intermediate Hi-Lo tube ETT without suction; ETT – endotracheal tube.

**Figure 2:**
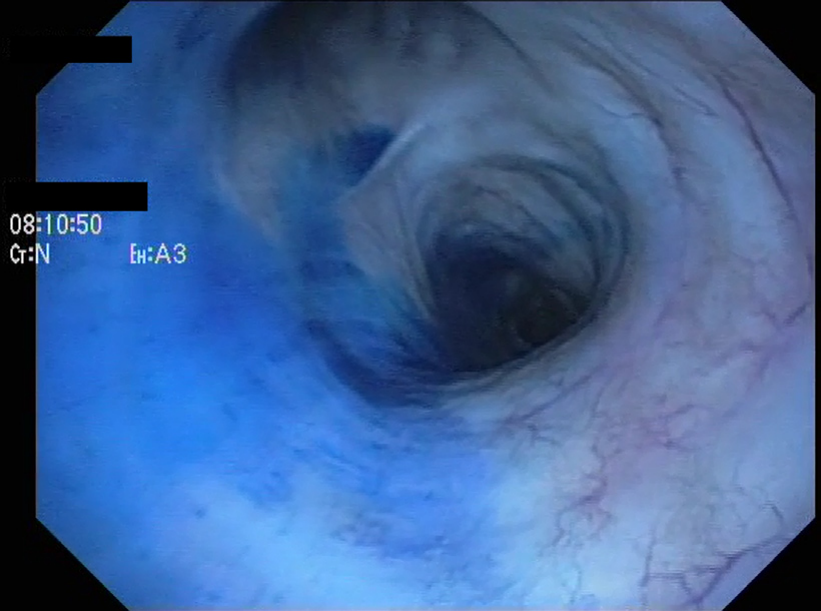
Distribution of methylene blue dye in the distal trachea and bronchi after the cuff deflation.

Immediately before tracheal extubation, there was no difference in the amount of dye below the vocal cords and above the inflated cuff (Table 5). Upon cuff deflation, dye migrated more into the distal trachea in 56% of GStand, 13% of GEvac patients, and 29% of GISIS patients (*P* = 0.045) (Table 5). Dye migrated below the carina and into the bronchi in three GStand patients, one GISIS patient, and no GEvac patients (*P* = 0.265) (Table 5). Figure 2 illustrates a typical view of dye that passed down the distal trachea into the bronchial lumen. Mild to moderate amounts of secretions were present within the lumen of ETT in the majority of patients. Secretions typically accumulated in the proximal trachea above the inflated cuff and in the distal trachea adjacent to the ETT tip. No secretions were observed below the carina in about two-thirds of patients (Table 5). There was no difference in the amount of secretions among groups just before tracheal extubation.

**Table 5:** Extubation bronchoscopy data*^†^

Grade	GISIS N = 17	GEvac N = 17	GStand N = 16	P value
Dye in the proximal trachea below vocal cords during and after ETT cuff deflation				0.412
0	2	0	0	
1	7	7	5	
2	7	5	9	
3	0	1	1	
Dye within the distal trachea during and after ETT cuff deflation				0.045
0	12	13	7	
1	4	2	4	
2	1	0	5	
3	0	0	0		
Dye below the carina and in the bronchi during and after ETT cuff deflation				0.265
0	16	15	13	
1	1	0	1	
2	0	0	2	
3	0	0	0	
Ischemia below the vocal cords				0.415
0	4	0	2	
1	4	7	6	
2	6	3	6	
3	2	2	1	
Blood below the vocal cords				0.546
0	5	3	7	
1	7	8	5	
2	4	2	2	
3	0	0	0	
Secretions within the ETT lumen				0.186
0	1	1	0	
1	4	6	4	
2	11	4	7	
3	0	4	4	
Secretions below the carina and bronchi				0.400
0	9	12	10	
1	4	1	5	
2	3	2	1	
3	0	0	0	

*Abbreviations: GISIS – Teleflex ISIS HVT tube; GEvac – Mallinckrodt TaperGuard Evac tube; GStand –Mallinckrodt Intermediate Hi-Lo tube ETT without suction; ETT – endotracheal tube.

†Some videos could not be evaluated due to too quick extubation.

All patients had satisfactory oxygenation and ventilation after one hour in the PACU. Three GISIS, 2 GEvac, and 4 GStand patients developed cough and sputum production in the 24-hour period after anesthesia. One additional GEvac patient developed bronchospasm that required treatment. The maximum respiratory rate, temperature, SaO_2_, and use of supplemental oxygen at 24 hours postoperatively were the same in all groups. No adverse events were related to the use of methylene blue dye.

## DISCUSSION

In this pilot study, ETT with CASS were efficient in removing secretions from above the inflated cuff and in preventing secretions from passing below the cuff during general anesthesia. The ETT with CASS also significantly reduced the incidence of secretions reaching the distal trachea and bronchi during cuff deflation and tracheal extubation. Frequent FOB revealed unexpected dynamic movement of secretions and migration of instilled dye. Patients undergoing abdominal surgery lasting longer than two hours were selected because they have a relatively high incidence of PPC, and overall the largest number of PPC occurs after abdominal surgery (2). We compared two different ETT with CASS because an *in vitro* study showed that ETTs with CASS have different cuff and suction port designs (6).

More patients from the GStand group had dye present in the trachea above the cuff than the other two groups with ETT and CASS. This could mean that both ETT with CASS effectively removed secretions, and/or that their bulkier size (outer diameter greater than that of standard ETT) prevented dye from entering the trachea through the glottis inlet. Moreover, in several instances we needed to stop giving dye to the GStand patients because of overflow conditions. Although the GStand patients received less dye on average, they had more dye present in the trachea.

Consistent with our results, D’Haese et al (7) showed that only the barrel cuff of the standard ETT leaked methylene blue into the distal trachea. In our study, dye migrated through folds in the standard ETT cuff starting about three hours after intubation, despite a continuous cuff pressure of 20 mm Hg. No dye leaked past the inflated cuff of any ETT with CASS probably because of advanced cuff design. But D’Haese et al did not evaluate the final disposition of the dye after cuff deflation and tracheal extubation. We found that when the cuff was deflated, in 56% of GStand patients, dye passed into the distal trachea. Dye migrated less frequently into the distal trachea of the GISIS patients (29%) compared with GEvac patients (12%). It passed down into the bronchi in 19% of GStand patients, 6% of the GISIS patients, and none of the GEvac patients. These observations suggest that suction ETT with CASS prevented the majority of secretions and dye from reaching the distal trachea and bronchi and significantly reduced the distal migration of dye after cuff deflation. The clinical significance of micro-aspiration during general anesthesia for abdominal surgery is unknown. An appropriately powered prospective clinical trial is required to determine whether the management of surgical patients in the operating room with suction ETT and CASS would decrease the incidence and severity of PPC.

**Figure 3:**
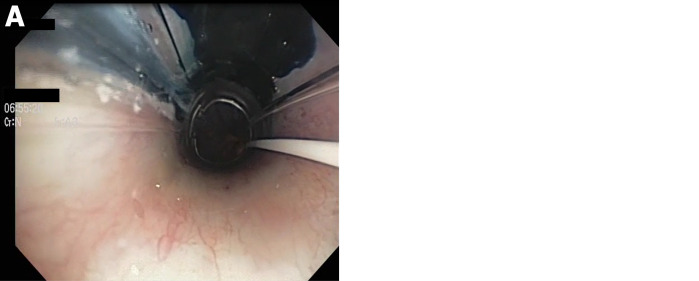
**(A)** Initial distribution of methylene blue dye in the trachea above the cuff. (**B)** Later distribution of methylene blue dye in the trachea above the cuff.

In all three ETT types, dye and secretions migrated from the hypopharynx through a passage between the ETT shaft and the anterior portion of the vocal cords along the path of least resistance. The path for dye migration was usually at the 12 o’clock position rather than the dependent 6 o’clock position (Figure 3A and 3B). The soft tissues of the epiglottis, glottis, and vocal cords in paralyzed patients formed a relatively efficient mechanical barrier around the shaft of the ETT, preventing the passage of secretions. The dye did not migrate from the pharynx into the trachea if this space was completely closed off by adjacent soft tissue (1 GISIS, 1 GEvac, and 1 GStand patient). After reversal of neuromuscular blockade and return of spontaneous ventilation, dye passed through the vocal cords into the trachea above the inflated cuff in all patients except two GISIS patients. These findings should be taken into account when microaspiration studies from ICU or operating room patients are compared. Spontaneous ventilation, coughing, and bucking may significantly affect the incidence and volume of microaspiration into the distal airway and bronchi, especially during emergence from general anesthesia and tracheal extubation.

We also observed that the application of negative suction pressure within the closed subglottic space would cause the soft tissue to collapse. Secretions and dye that entered the suction port and suction channel would stop. This limitation could be overcome by modification of the suction ETT design with a separate channel and port that allows atmospheric air to enter the trachea lumen below the vocal cords and above the inflated ETT cuff. Also, after intubation, the suctioning port of ETT was typically not located in the dependent 6 o’clock position. The port was usually in the 7 or 8 o’clock position, and in some cases the 9 o’clock position, although the ETT was taped with the posterior marking line exactly in the 6 o’clock position. The position was checked immediately after intubation and was not changed throughout the duration of anesthesia.

Since we performed FOB every 20 minutes, we could follow the dynamic movement of secretions that appeared below the ETT cuff during general anesthesia. Secretions appeared in the trachea below the vocal cords and above the cuff in 78% of the patients, while they appeared in the bronchi distal to the carina in 38% of the patients. Almost all of the patients (98%) accumulated secretions within the ETT lumen. This suggests secretions were commonly produced in the trachea where the ETT was compressing and irritating the tracheal mucosa, rather than migrating from the distal bronchi into the trachea lumen. The interesting observation was that under positive pressure ventilation, distal tracheal secretions would not migrate distally into the bronchi but rather would migrate upward into the ETT lumen.

Only one patient in each group developed post-operative cough, sputum production, or wheezing within 24 hours and no patient developed a severe PPC (pneumonia, aspiration pneumonitis, atelectasis, pleural effusion, or respiratory insufficiency/failure). This pilot study did not show a difference in PPC between patients managed with an ETT with CASS vs a standard ETT. Although microaspiration leads to pulmonary complications in the ICU, it is unknown if aspiration of secretions past an inflated ETT cuff leads to PPC following prolonged general anesthesia. An adequately powered prospective randomized clinical trial is required to determine whether prevention of microaspiration using a subglottic suction ETT with CASS during prolonged general anesthesia decreases the incidence of PPC, hospital length of stay, cost, and mortality.

Several methods were proposed to evaluate microaspiration in the ICU: radioactive labeling of enteral feedings, measurement of pepsin in the tracheal aspirate, and using a marker such as methylene blue to visualize secretions with fiberoptic bronchoscopy (8). We used methylene blue visualization because of familiarity with FOB, simplicity of instillation, and safety of methylene blue. We observed no methylene blue side effects. The dose used in our study was significantly lower than the dose used in gastrointestinal studies, or even IV dose (1 mg/kg). Moreover, methylene blue in is not absorbed by non-absorptive mucosa such as oral squamous cell mucosa or the gastric epithelium. Therefore, the most of it is suctioned out from the tracheal and bronchial mucosa with minimal resorption.

The study suffers from several limitations. First, we could not determine the actual amount of patient secretions present in the trachea because some secretions and dye traveled down the esophagus into the stomach. Second, our results are restricted to surgical patients managed with general anesthesia and muscle paralysis. Dye passed more easily through the vocal cords into the trachea after reversal of muscle paralysis and during emergence from general anesthesia when the patients were breathing spontaneously, coughing, and swallowing. Third, the study was underpowered to show the deference in PPC among groups. It was a pilot study with a low number of patients. Moreover, we studied relatively healthy patients and had a limited follow-up.

In conclusion, a large prospective randomized clinical trial is required to determine whether reducing the amount of micro-aspiration into the trachea and bronchi during general endotracheal anesthesia decreases the incidence and severity of post-operative pulmonary complications.
